# Supplementary material for: Transition from the topological to the chaotic in the nonlinear Su–Schrieffer–Heeger model
Source: Nat Commun. 2025 Jan 29;16:422. doi: 10.1038/s41467-024-55237-3 (PMC11779912; doi:10.1038/s41467-024-55237-3)
Supplement: Supplementary file 1 — Supplementary Information [file 41467_2024_55237_MOESM1_ESM.pdf]

Supplementary Information

Transition from the topological to the chaotic in the nonlinear Su-Schrieffer-Heeger model

Sone et al.

Supplementary Note 1 - Possible optical setup of the nonlinear SSH model.

The nonlinear SSH model (Eqs. (4) and (5) in the main text) can describe various systems, such as topological photonics and electrical circuits. We here discuss the possible realization of the nonlinear coupling used in the model by using photonic systems. Specifically, we consider an interaction between two sites via an optical fiber used in Ref. [1]. When one uses such an optical fiber, the phase of the coupling depends on the amplitude of the light because the light propagating in the optical fiber is affected by the Kerr nonlinearity, and thus the phase shift occurs depending on its amplitude. Thus, the coupling term has a form of $ce^{i\kappa|\Psi_j|^2}\Psi_j$ with c being the strength of the coupling and κ being the coefficient of the Kerr nonlinearity. Remaining the leading order terms, we obtain $c(1+i\kappa|\Psi_j|^2-\kappa^2|\Psi_j|^4/2+\cdots)\Psi_j$. The second term has a similar form to the nonlinear coupling in the nonlinear SSH model except for the multiplication of the imaginary unit i. The third term is a higher–order term without the imaginary unit and thus can induce the nonlinearity–induced topological phase transition as in the models discussed in the main text.

Supplementary Note 2 - Lyapunov exponents

Lyapunov exponents are the rate of the amplification or attenuation of the perturbation from the solution of a nonlinear dynamical system, whose positivity indicates the chaos of the dynamical system. We numerically confirm the bifurcation to chaos by calculating the Lyapunov exponent of the dynamical system describing zero modes in the nonlinear SSH model (Eq. (7) in the main text). One can calculate the Lyapunov exponent of a one–dimensional discrete dynamical system $\psi_{i+1} = F(\psi_i)$ such as Eq. (7) in the main text from the following formula:

$$\lambda = \lim_{L\to\infty} \frac{1}{L} \sum_{i=1}^{L} \log |F'(\tilde{\psi}_i)|, \tag{S1}$$

where F' is the derivative of F by ψ_i and $\tilde{\psi}_i$ $(i = 1, \cdots, L)$ is an orbit of the dynamical system $\psi_{i+1} = F(\psi_i)$.

Supplementary Figure 1 shows the numerically obtained Lyapunov exponents of the dynamical system describing zero modes in the nonlinear SSH model. We use $L = 10000$ and $b = c = d = 1$. Around $a = 2.3$, the Lyapunov exponent becomes positive, which indicates the bifurcation to chaos. We estimate the chaos transition point in Fig. 2 in the main text from this numerical result. As is usually seen in chaotic maps, even larger a than the chaos

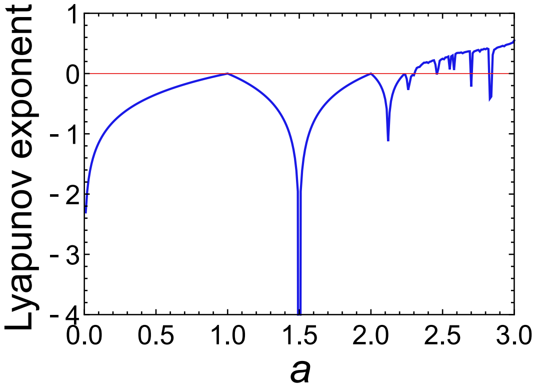

Supplementary Figure 1. **Lyapunov exponents of the nonlinear SSH model.** We fix the parameters $b = c = d = 1$ and change the parameter a. We calculate $L = 10000$ steps of the dynamical system of zero modes in the nonlinear SSH model. The blue curve shows the numerically obtained Lyapunov exponents. The Lyapunov exponents above the red line are positive, which indicates the chaos of the dynamical system.

transition point, Lyapunov exponents can be negative in some parameter regions. However, in these parameter regions, the steady–state solutions are periodic and thus still break the bulk–edge correspondence.

One can also find zero Lyapunov exponents at some a's. Such a's correspond to the period–doubling bifurcation points. In particular, $a = 1$ and $a = 2$ are the bifurcation points as presented in Fig. 2 in the main text. Zero Lyapunov exponents at $a = 1$ and $a = 2$ can be analytically confirmed from the fact that the corresponding fixed points are linearly marginal in the spatial dynamics (Eq. (7) in the main text), i.e., the derivative of F is zero at the fixed points: $|F'(a = 1, \tilde{\psi}_i = 0)| = 0$ and $|F'(a = 2, \tilde{\psi}_i = 1)| = 0$.

Supplementary Note 3 - Temporal linear stability and instability of zero modes. While we have analyzed the stability of the spatial dynamics of zero modes in the previous section, we also conduct the linear stability analysis of the temporal dynamics of zero modes. To this end, we consider the linearization of Eqs. (4) and (5) in the main text around the

periodic solution $\Psi^0_{A,B}(x) = \psi_{A,B}e^{ikx}$. We obtain the linearized dynamics,

$$
\begin{aligned}
i\frac{\partial}{\partial t}\begin{pmatrix}\delta\psi_A\\ \delta\psi_B\\ \delta\psi_A^*\\ \delta\psi_B^*\end{pmatrix} &= H_L(\psi_A,\psi_B)\begin{pmatrix}\delta\psi_A\\ \delta\psi_B\\ \delta\psi_A^*\\ \delta\psi_B^*\end{pmatrix}\\
&= \begin{pmatrix} b\psi_A^*\psi_B & \tilde{a}b|\psi_B|^2 & b\psi_A\psi_B & b\psi_B^2\\ \tilde{a}^*+b|\psi_A|^2 & b\psi_A\psi_B^* & b\psi_A^2 & b\psi_A\psi_B\\ -b\psi_A^*\psi_B^* & -b\psi_B^{*2} & -b\psi_A\psi_B^* & -\tilde{a}^*-b|\psi_B|^2\\ -b\psi_A^{*2} & -b\psi_A^*\psi_B^* & -\tilde{a}-b|\psi_A|^2 & -b\psi_A^*\psi_B \end{pmatrix}\begin{pmatrix}\delta\psi_A\\ \delta\psi_B\\ \delta\psi_A^*\\ \delta\psi_B^*\end{pmatrix},
\end{aligned}
\quad \text{(S2)}
$$

where we consider a periodic modulation $\Psi_{A,B}(x) = \Psi^0_{A,B}(x) + \delta\psi_{A,B}e^{ik'x}$ and $\tilde{a} = a - b(|\psi_A|^2 + |\psi_B|^2) + de^{ik'}$. We note that the wavenumber of this periodic modulation can be different from that of the periodic solution $\Psi^0_{A,B}(x)$.

By calculating the eigenvalues of $H_L(\psi_A, \psi_B)$, we find that the bifurcation in the nonlinear dynamical system in the spatial direction (Eq. (7) in the main text) is also related to the temporal instability of zero modes. We here consider the periodic solution corresponding to bulk modes with zero eigenvalues: $k = \pi$, $(\psi_A, \psi_B) = (w/\sqrt{2}, \pm w/\sqrt{2})$, $w = a - 1$. Then, the eigenvalues of $H_L(\psi_A, \psi_B)$ become $\lambda_{1\pm} = \pm\sqrt{|a + de^{ik'}|^2 + 2bw(a + d\cos k') + (bw)^2}$ and $\lambda_{2\pm} = \pm\sqrt{|a + de^{ik'}|^2 + 4bw(a + d\cos k') + 3(bw)^2}$. $\lambda_{1\pm}$ are always real, while $\lambda_{2\pm}$ become purely imaginary if $|a + de^{ik'}|^2 + 4bw(a + d\cos k') + 3(bw)^2$ is negative. Since $|a + de^{ik'}|^2 + 4bw(a + d\cos k') + 3(bw)^2$ takes minimum at $k' = 0$ or $k' = \pi$ and becomes zero at $k' = \pi$, $\lambda_{2\pm}$ are real if $(a + d)^2 + 4bw(a + d) + 3(bw)^2$ is positive. In the case of negative b, we obtain the condition for the linear instability, i.e., imaginary $\lambda_{2\pm}$ as $(a+d)/(3|b|) < w < (a+d)/|b|$. When we assume the parameters $a > 0$, $b = -1$, and $d = 1$ as in Fig. 2 in the main text, one can confirm that this inequality is equivalent to $a > 2$, at which the localized zero mode becomes unstable in the spatial dynamics in Eq. (7) in the main text. Thus, the spatial and temporal instability of zero modes are closely related to each other.

Supplementary Note 4 - Bulk–edge correspondence in finite systems

We here numerically confirm the bulk–edge correspondence in finite systems of the nonlinear SSH model (Eqs. (4) and (5) in the main text). To judge the localization or anti–localization

of zero modes, we define the indicator of localization P as

$$P = w_{\mathrm{edge}}/w_{\mathrm{second}}, \tag{S3}$$

$$w_{\mathrm{edge}} = \max\left(\sum_j \Psi_j(1), \sum_j \Psi_j(L)\right), \tag{S4}$$

$$w_{\mathrm{second}} = \begin{cases} \sum_j |\Psi_j(2)|^2 & \sum_j |\Psi_j(1)|^2 > \sum_j |\Psi_j(L)|^2 \\ \sum_j |\Psi_j(L-1)|^2 & (\text{otherwise}) \end{cases}, \tag{S5}$$

with L being the system size. $\Psi_j(x)$ is the nonlinear eigenvector of a zero mode and x and j represent the location and the internal degrees of freedom, respectively. This localization indicator becomes larger (smaller) than one, when the zero mode is localized (anti–localized) at the edge. We note that one should modify the definition of the localization indicator in long–range models, while we here focus on the nonlinear SSH model whose hopping range is one.

We numerically solve the nonlinear eigenvalue problem of the finite chains of the nonlinear SSH model with different system sizes $L = 5$, 10, 15, and 20. To solve the nonlinear eigenvalue problem, we assume the eigenequation as an algebraic equation and use the quasi–Newton method [2]. Supplementary Figure 2 shows the minimum absolute values of eigenvalues and the localization indicators (Supplementary Eq. (S3)) of the corresponding eigenvectors. At $a = 1.5$ (Supplementary Fig. 2a), the nonlinear winding number is nonzero $\nu_{\mathrm{NL}} = 1$ in the range of amplitude, $0.5 < w < 2.5$. In this regime, we obtain localized zero modes corresponding to the nonzero winding number. Meanwhile, we obtain anti–localized zero modes at $w < 0.5$ and no zero modes at $w > 2.5$. Therefore, the nonlinear winding number predicts the existence or absence of the localized zero modes at $a = 1.5$. We can also confirm the bulk–edge correspondence at $a = 1.9$ as shown in Supplementary Fig. 2b.

In contrast, if we consider $a = 2.1$, we obtain anti–localized zero modes even in the topologically nontrivial phase at $w > 1.1$ (Supplementary Fig. 2c). Therefore, the bulk–edge correspondence is broken in this parameter regime, which is consistent with the result in the semi–infinite system shown in Fig. 2 in the main text. We note that the zero modes in the semi–infinite system exhibit the localization indicator $P > 1$ even at $a > 2$ and thus can be regarded as locally localized but globally anti–localized zero modes. Meanwhile, the zero modes in finite systems can be locally anti–localized in the sense that the localization indicator P is smaller than one. While both the breakdowns of the bulk–edge correspondence

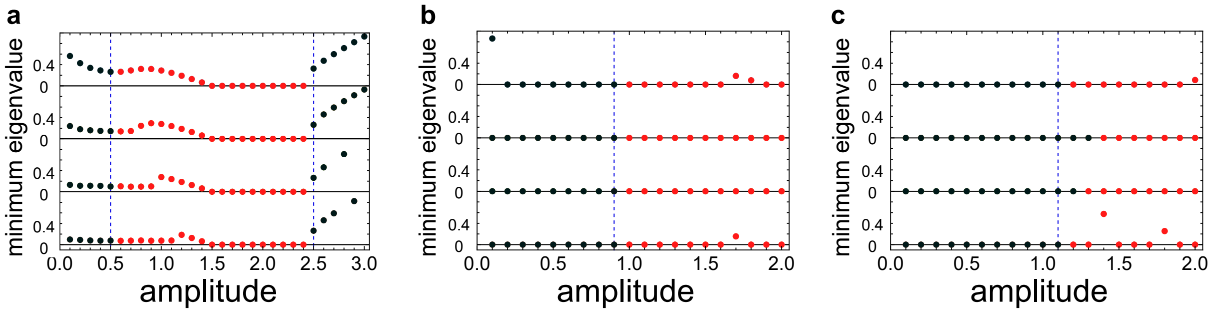

Supplementary Figure 2. **Minimum absolute values of eigenvalues and localization indicators P of the finite system of the nonlinear SSH model.** We fix the parameters $b = c = d = 1$. We plot the minimum absolute values of the nonlinear eigenvalues at the system size $L = 5$, 10, 15, and 20 from the top. The red (black) circles represent the localization $P > 1$ (anti–localization $P < 1$) of the corresponding nonlinear eigenvectors. **a** Localization indicators at $a = 1.5$. The blue dashed lines are the transition point of the nonlinear winding number. We can confirm the bulk–edge correspondence between the nonlinear winding number and the localized zero modes. We note that some data points disappear at $a > 2.5$ due to the difficulty of the convergence of the numerical techniques under the strong nonlinearity. **b** Localization indicators at $a = 1.9$. The localization and anti–localization of zero modes are switched at the transition point represented by the blue dashed line, which indicates the bulk–edge correspondence. **c** Localization indicators at $a = 2.1$. Even at the amplitude larger than the critical amplitude $w = 1.1$ (the blue dashed line), there exist anti–localized zero modes. Therefore, the bulk–edge correspondence is broken in this parameter regime, which is consistent with the analysis in the semi–infinite system.

observed in finite and semi–infinite systems are related to the period–doubling bifurcation, the detailed mechanism of the anti–localization of zero modes in finite systems remains a future issue.

The obtained nonlinear eigenvalues are not exactly zero due to the finite–size effect. To confirm the convergence of nonlinear eigenvalues to zero in the thermodynamic limit $L \to \infty$, we investigate the finite–size scaling of the minimum absolute values of nonlinear eigenvalues. Supplementary Figure 3a shows the minimum absolute values of nonlinear eigenvalues at different system sizes. We here use the parameters $a = 1.5$, $b = c = 1$, and $d = 1$, and fix the edge amplitude to be $w_{\mathrm{edge}} = 0.6$. We confirm that the nonlinear eigenvalues are inversely proportional to the system size and thus converge to zero in the thermodynamic limit. This

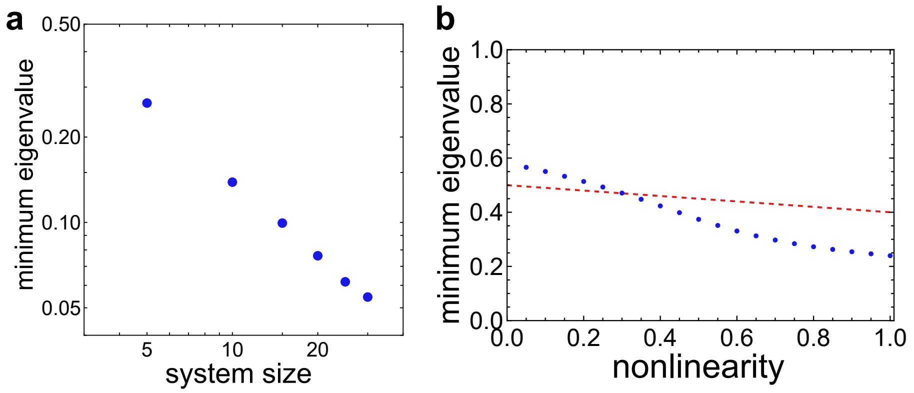

Supplementary Figure 3. **Size– and nonlinearity–dependence of the minimum absolute values of nonlinear eigenvalues.** **a** Finite-size scaling of the minimum absolute values of nonlinear eigenvalues in the nonlinear SSH model. We use the parameters $a = 0.6$ and $b = c = d = 1$. We confirm that the minimum eigenvalues are inversely proportional to the system size and thus converge to zero in the thermodynamic limit. **b** Disappearance of the anti–localized zero mode in the linear limit. The red dashed line shows the bulk band gap obtained from the Bloch ansatz. The horizontal axis shows the value of b (we set $b = c$). We fix the other parameters as $a = 1.5$, $d = 1$, and the amplitude $w = 0.1$. At $b = 0.3$, the minimum absolute values of the nonlinear eigenvalues become larger than the bulk band gap. Thus, one cannot distinguish the anti–localized modes and bulk modes at $b < 0.3$, which implies the disappearance of the anti–localized zero modes in the linear limit.

system–size dependence of the nonlinear eigenvalue is different from the linear case, which is exponentially decreased in the system size. This is because the nonlinearity–induced edge modes have nonvanishing amplitudes far from the edge $x \to \pm\infty$, and the left and right localized modes Ψ_{left}, Ψ_{right} have a larger resonance integral than in linear cases. In fact, the resonance integral becomes

$$\frac{\sum_{x=1}^{L}\sum_{j=A,B}\Psi_{\text{left},j}(x)^{*}\vec{f}(\Psi_{\text{right},j}(x))}{\sqrt{\sum_{x=1}^{L}\sum_{j=A,B}\Psi_{\text{left},j}(x)^{*}\Psi_{\text{left},j}(x)}\sqrt{\sum_{x=1}^{L}\sum_{j=A,B}\Psi_{\text{right},j}(x)^{*}\Psi_{\text{right},j}(x)}} = \frac{\mathcal{O}(1)}{\mathcal{O}(L)} = \mathcal{O}(L^{-1}), \tag{S6}$$

where $\vec{f}$ represents the right–hand side of Eqs. (4) and (5) in the main text. To obtain the

system–size dependence, we utilize the fact that $\vec{f}(\Psi_{\text{right},j}(x))$ is zero in the bulk, and thus

$$\sum_{x=1}^{L}\sum_{j=A,B}\Psi_{\text{left},j}(x)^{*}\vec{f}(\Psi_{\text{right},j}(x))=\sum_{j=A,B}\Big[\Psi_{\text{left},j}(1)^{*}\vec{f}(\Psi_{\text{right},j}(1))+\Psi_{\text{left},j}(L)^{*}\vec{f}(\Psi_{\text{right},j}(L))\Big] \tag{S7}$$

becomes almost independent of the system size. Thus, the interaction between the left and right localized modes is also proportional to the inverse of the system size, which results in the power–law system–size dependence of the nonlinear eigenvalue of edge modes. We note that similar size dependence is observed in a previous paper on the nonlinear Chern number [2].

It is also noteworthy that the anti–localized zero modes disappear in the linear limit $b, c \to 0$. We consider the case of $b = c$ and numerically confirm such disappearance of the anti–localized zero modes by calculating the nonlinear eigenvalues at different b. Supplementary Figure 3b shows the minimum absolute values of the nonlinear eigenvalues and the size of the bulk band gaps predicted from the Bloch ansatz. At sufficiently small b, the nonlinear eigenvalues become larger than the bulk band gaps, and thus one cannot distinguish the anti–localized zero modes and bulk modes. Therefore, anti–localized zero modes are induced by the nonlinearity and disappear in the linear limit.

While we have used the localization indicator P (Supplementary Eq. (S3)) in the above numerical calculations, there are other possible definitions of the localization indicators. Specifically, we can consider the following definitions that distinguish the localization and anti–localization of zero modes $\Psi_j(x)$ in a global sense: (1) $P_1 = w_{\text{edge}}/w_{\text{ave}}$ with $w_{\text{ave}} = \sum_{x,i}|\Psi_i(x)|^2/L$ being the averaged amplitude, (2) $P_2 = w_{\text{edge}}/w(x)$ with $w(x) = \sum_i |\Psi_i(x)|^2$ being the amplitude and x denoting a bulk site that we determine in hand, (3) $P_3 = w_{\text{edge}}/w_{\text{max}}$ with $w_{\text{max}} = \max_x\left(\sum_i |\Psi_i(x)|^2\right)$ being the maximum amplitude. Unfortunately, these localization indicators can be inconsistent with the nonlinear winding number at $a < 2$, where the bulk–edge correspondence still holds true in the nonlinear SSH model. Supplementary Figure 4 shows the localization indicators of the nonlinear eigenvectors with the minimum absolute values of nonlinear eigenvalues. At $a = 1.5$, all the indicators correspond to the localization and anti–localization of zero modes predicted from the nonlinear winding number. In contrast, at $a = 1.9$, the indicators do not correspond to the nonlinear winding number. In this parameter regime, the bulk–edge correspondence is confirmed in semi–infinite systems (Fig. 2 in the main text). Therefore, the localization indicators P_1, P_2,

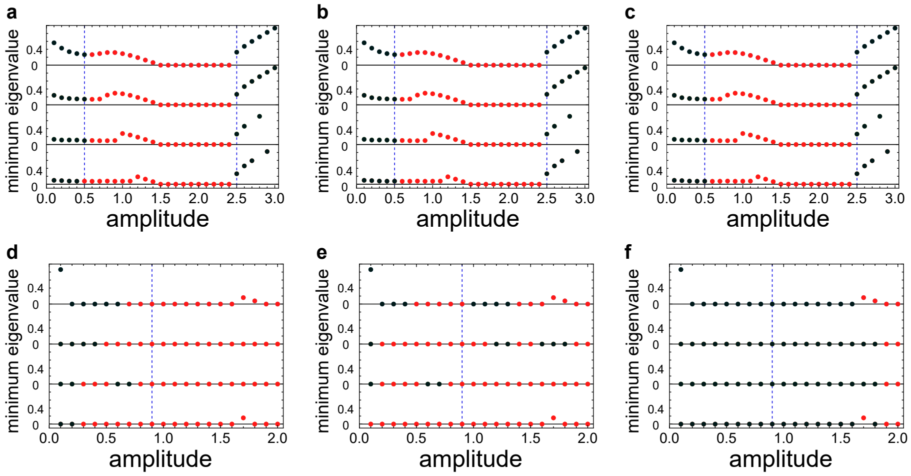

Supplementary Figure 4. **Minimum absolute values of eigenvalues and different definitions of localization indicators $P_{1,2,3}$ of the finite system of the nonlinear SSH model.** We fix the parameters $b = c = -1$ and $d = 1$. We plot the minimum absolute values of the nonlinear eigenvalues at the system size $L = 5$, 10, 15, and 20 from the top. The red (resp. black) circles represent $P_{1,2,3} > 1$ (resp. $P_{1,2,3} < 1$) of the corresponding nonlinear eigenvectors. **a-c** Localization indicators at $a = 1.5$. Panels **a**, **b**, and **c** show the localization indicators P_1, P_2, and P_3, respectively. The blue dashed lines are the transition point of the nonlinear winding number. We can confirm the bulk–edge correspondence between the nonlinear winding number and the localized zero modes. We note that some data points disappear at an amplitude larger than 2.5 due to the difficulty of the convergence of the numerical techniques under the strong nonlinearity. In this parameter region, all the indicators correspond to the bulk topological invariants. **d-f** Localization indicators at $a = 1.9$. Panels **d**, **e**, and **f** show the localization indicators P_1, P_2, and P_3, respectively. The blue dashed lines show the transition point of the nonlinear winding number, while the localization indicators are unchanged at those boundaries. Since the bulk–edge correspondence should still be valid in this parameter region as inferred from the bifurcation diagram in Fig. 2 in the main text, the localization indicators $P_{1,2,3}$ are useless to discuss the bulk–boundary boundary correspondence in finite systems.

and P_3 are unsuitable to discuss the bulk–edge correspondence in finite nonlinear systems.

Supplementary Note 5 - Robustness of edge modes and chaos transitions against spatial disorders

We here confirm that topological edge modes and their chaos transitions are robust against disorders in the nonlinear SSH model. For simplicity, we consider the spatial inhomogeneity of a and d and fix the other parameters $b = c = -1$ independently of the location. We note that if we introduce disorders in b and c, we can renormalize their effects into those of a and d. We introduce the disorder in a, d by setting $a(x)$, $d(x)$ at each location x as $(1 + \Delta a(x))a_{\mathrm{ave}}$, $(1 + \Delta d(x))d_{\mathrm{ave}}$, where $\Delta a(x)$ and $\Delta d(x)$ are randomly determined from a uniform distribution $[-\delta, \delta]$. We fix the expectation value of $d(x)$ as $d_{\mathrm{ave}} = 1$. We calculate the spatial dynamics similar to Eq. (7) in the main text at $\delta = 0.01$ and 0.05.

Supplementary Figure 5 shows the bifurcation diagram at each strength of the disorder. At $a_{\mathrm{ave}} < 2$, the convergent behavior to $|\Psi_A(x)| \sim \sqrt{(a-b)/d}$ seems to remain if we permit the fluctuation of $\Psi_A(x)$ comparable to that of $a(x)$. We find that the period–doubling bifurcations disappear at large disorder δ. At large a_{ave}, the spatial dynamics becomes very noisy, which implies the spatial chaos of zero modes.

We also calculate the Lyapunov exponents under the existence of disorder (Supplementary Fig. 5c,d) as in Supplementary Fig. 1. We confirm that the Lyapunov exponent becomes positive at a certain a_{ave}, and thus the spatial dynamics exhibits a chaos transition. Therefore, the chaos transition and the associated breakdown of the bulk–edge correspondence are also robust against disorders. We note that the periodic solutions after the chaos transition, i.e., the windows of chaos disappear under the existence of disorder. Such disorder–induced chaos is consistent with previous research [3] on the discrete nonlinear dynamics with noises.

In some nonlinear models, eigenvalues of topological edge modes can be shifted from zero even without disorders [4]. In contrast, the present result indicates that the nonlinear eigenvalue of edge modes in the nonlinear SSH model is zero and immune to disorders and nonlinearity. This is because of the sublattice symmetry (Eq. (18) in Methods), which guarantees the symmetry of the band structure and the zero eigenvalue of edge modes (at least in the linear case).

Supplementary Note 6 - Stability of edge modes in nonlinear topological mechanics.

While we have discussed the destabilization of edge modes by chaos transitions in the main text, some nonlinear topological insulators do not exhibit such chaos transitions at arbitrary strength of the nonlinearity. We here analyze nonlinear topological mechanics composed of

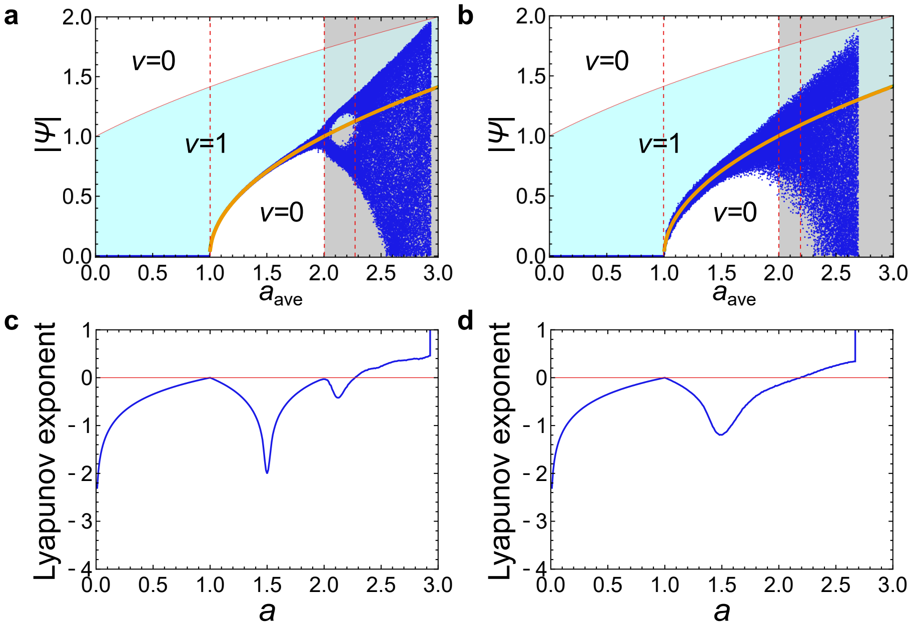

Supplementary Figure 5. **Bifurcation plots and Lyapunov exponents under the existence of disorder.** **a,b,** Bifurcation plots under the existence of disorder. We fix the parameters $b = c = -1$ and $d_{\text{ave}} = 1$, and change a_{ave}. We set the strength of disorder as $\delta = 0.01$ in panel **a** and $\delta = 0.05$ in panel **b**. The red lines at $a_{\text{ave}} = 1$ and $a_{\text{ave}} = 2$ represent the bifurcation points in the disorder-free case. The third red line shows the chaos transition point obtained from the numerical calculation of the Lyapunov exponents in panels **c** and **d**. We obtain almost convergent solutions at $a_{\text{ave}} < 2$, which indicates the robustness of topological edge modes against disorders. One can confirm the period–doubling bifurcation around $a_{\text{ave}} \sim 2$ in panel **a**, while the other period–doubling bifurcations seen in the disorder–free case disappear. We note that at large a_{ave}, the spatial dynamics diverge to infinity, and thus there are no data points. **c,d,** Lyapunov exponents under the existence of disorder. We use the same parameters as in panels **a** and **b** and set the strength of disorder as $\delta = 0.01$ in panel **c** and $\delta = 0.05$ in panel **d**. For both strengths of disorders, we obtain positive Lyapunov exponents (above the red lines) at any larger a_{ave} than critical values, which indicates the robustness of the chaos transition against disorder. At large a_{ave}, since the spatial dynamics diverge to infinity, the Lyapunov exponents also diverge.

elliptic gears that are studied in Ref. [5], and show that the absence of chaos transitions is related to the linear stability in the spatial dynamics. The dynamics of that nonlinear

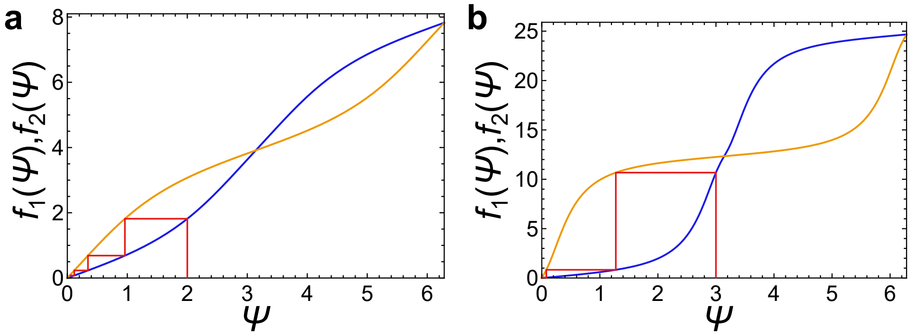

Supplementary Figure 6. **Cobweb plots of the spatial dynamics of a topological mechanics.** We present the cobweb plots of the spatial dynamics in Supplementary Eq. (S12). The orange (resp. blue) curves show $y = f_\epsilon(x)$ (resp. $y = f_{-\epsilon}(x)$). The red polylines represent the spatial dynamics. If we start from $\Psi_A(1) < \pi$, $\Psi_A(x)$ must be converged to zero at any e, which indicates the parameter independence of the stability of the fixed point at $\Psi_A(x) = 0$. We fix the parameter $a = 1$ and use $\epsilon = 0.5$ in panel **a** and $\epsilon = 0.9$ in panel **b**.

topological mechanics is described as follows:

$$i\partial_t \Psi_A(x) = f_\epsilon(\Psi_B(x)) - f_{-\epsilon}(\Psi_B(x-1)), \tag{S8}$$

$$i\partial_t \Psi_B(x) = f_\epsilon(\Psi_A(x)) - f_{-\epsilon}(\Psi_A(x+1)), \tag{S9}$$

where $\Psi_{A,B}(x)$ are the complex field variable. $f_\epsilon(\Psi_{A,B}(x))$ is the nonlinear function defined as

$$f_\epsilon(\Psi) = s_\epsilon(\operatorname{Re}\Psi) + i s_\epsilon(\operatorname{Im}\Psi), \tag{S10}$$

$$s_\epsilon(x) = a(1-\epsilon^2)\int_0^x \frac{\sqrt{1+2\epsilon\cos x' + \epsilon^2}}{(1+\epsilon\cos x')^2} dx', \tag{S11}$$

with a being the semi major–axis of the elliptic gears and ϵ being their eccentricity.

As in Eq. (7) in the main text, we focus on the zero nonlinear eigenvectors and derive the nonlinear dynamics describing their spatial distribution. Assuming $\Psi_B(x) = 0$, one can determine $\Psi_A(x+1)$ from $\Psi_A(x)$ so that they satisfy

$$f_\epsilon(\Psi_A(x)) = f_{-\epsilon}(\Psi_A(x+1)). \tag{S12}$$

Such spatial dynamics are captured by cobweb plots in Supplementary Fig. 6. Fortunately, the value of $\Psi_A(x+1)$ is uniquely determined at any parameters.

We investigate the spatial dynamics of zero modes at different ϵ and find that the fixed point at $\Psi_A(x) = 0$ is always stable unless the bulk topology is changed. We confirm this

from the fact that $\Psi_A(x+1)$ is smaller than $\Psi_A(x)$ for $\Psi_A(x) < \pi$ and $\epsilon > 0$. Therefore, the zero mode must be decayed to zero in the thermodynamic limit $x \to \infty$, which indicates the absence of the chaos transition. The situation is similar in other nonlinear models in, e.g., Ref. [5], where the nonlinearity–induced topological modes are linearly stable at arbitrary parameters.

Supplementary Note 7 - Linear stability analysis of the fixed points in the dynamical system describing zero modes of the extended nonlinear SSH model

While in the main text, we have numerically calculated the flow fields and demonstrated the correspondence between the nonlinear winding number and the dimension of the stable manifold in the dynamical system of zero modes, we can also calculate the dimension of the stable manifold from the linear stability analysis. Specifically, we consider the linearization around the fixed point Ψ_{fix} of the dynamical system in Eq. (9) in the main text and obtain

$$\begin{pmatrix} \delta\Psi_1(x+1) \\ \delta\Psi_1(x) \end{pmatrix} = \begin{pmatrix} -d/\alpha & -(a+3b|\Psi_{\text{fix}}|^2)/\alpha \\ 1 & 0 \end{pmatrix} \begin{pmatrix} \delta\Psi_1(x) \\ \delta\Psi_1(x-1) \end{pmatrix}, \tag{S13}$$

where $\delta\Psi = \Psi(x) - \Psi_{\text{fix}}$ is the deviation from the fixed point. Then, we calculate the eigenvalues $\lambda_\pm$ of the matrix in this equation. The dimension of the stable manifold is equal to the number of the eigenvalues that satisfy $|\lambda_\pm| < 1$.

Since the dynamical system in Eq. (9) in the main text can have three qualitatively different fixed points and limit cycles at $|\psi_{\text{fix}}|^2 = 0$, $(d-a-\alpha)/b$, and $(\alpha - a)/b$, we below calculate the linear stability of these fixed points. We note that the nonzero fixed points, $|\psi_{\text{fix}}|^2 = (d-a-\alpha)/b$ and $(\alpha-a)/b$, correspond to the topological edge modes that appear in the nonlinearity–induced topological phase because they converge to nonzero amplitude in the limit of $x \to \infty$. Below we focus on the parameter region $a, d, \alpha > 0$, while we can conduct a similar analysis in the cases of different choices of the signs of the parameters.

Stability analysis of the fixed point at $\psi_{\text{fix}} = 0$. The linearized matrix (Supplementary Eq. (S13)) around $\psi_{\text{fix}} = 0$ exhibits the eigenvalues

$$\lambda_\pm = \frac{-d \pm \sqrt{d^2 - 4\alpha a}}{2\alpha}. \tag{S14}$$

To investigate the linear stability of the fixed point at $\psi_{\text{fix}} = 0$, we separately consider the cases of $d^2 - 4\alpha a < 0$ and $d^2 - 4\alpha a > 0$.

First, if $d^2 - 4\alpha a$ is negative, the eigenvalues become a pair of complex conjugates. Then, we can calculate their absolute values as

$$|\lambda_\pm^2| = \frac{d^2 + (4\alpha a - d^2)}{(2\alpha)^2} = \frac{a}{\alpha}. \tag{S15}$$

Thus, one can determine the linear stability of the fixed point by comparing a and α. In the case of $a < \alpha$, the fixed point is linearly stable and has a two–dimensional stable manifold. In this parameter region, the winding number becomes $\nu_{\mathrm{NL}} = 2$, which corresponds to the dimension of the stable manifold. In contrast, when a is larger than α, the fixed point is fully unstable. Then, the winding number becomes $\nu_{\mathrm{NL}} = 0$, which is also equal to the dimension of the stable manifold.

Second, if $d^2 - 4\alpha a$ is positive, the eigenvalues $\lambda_\pm$ become real. Then, we can check that $|\lambda_-| < 1$ is equivalent to $d^2 - 4\alpha a < (2\alpha - d)^2$ and $2\alpha - d > 0$. We can also confirm that $|\lambda_+| > 1$ is equivalent to $d^2 - 4\alpha a < (d - 2\alpha)^2$ and $d - 2\alpha > 0$. The winding number becomes $\nu_{\mathrm{NL}} = 1$ in the parameter region $d > a + \alpha$. In this case, we can show

$$(d - 2\alpha)^2 = d^2 - 4\alpha d + 4\alpha^2 < d^2 - 4\alpha(a + \alpha) + 4\alpha^2 = d^2 - 4\alpha a, \tag{S16}$$

and thus confirm $|\lambda_-| > 1$ and $|\lambda_+| < 1$, which indicates that the stable manifold is one–dimension.. In contrast, if we consider the parameter region $d < a + \alpha$, we obtain $d^2 - 4\alpha a < (d - 2\alpha)^2$. Then, the sign of $2\alpha - d$ is determined by the sign of $\alpha - a$ as shown from the inequality

$$\alpha - a < 2\alpha - d < 2\sqrt{\alpha}(\sqrt{\alpha} - \sqrt{a}). \tag{S17}$$

Therefore, the dimension of the stable manifold is two in the case of $\alpha > a$ and zero in the case of $\alpha < a$. These results are consistent with the bulk–edge correspondence between the winding number and the dimension of the stable manifold.

Stability analysis of the fixed point at $|\psi_{\mathrm{fix}}|^2 = (d - a - \alpha)/b$. If we consider the parameter region $d < a + \alpha$, $\alpha < a$, and $b < 0$, we obtain the fixed point at $|\psi_{\mathrm{fix}}|^2 = (d - a - \alpha)/b$. This fixed point corresponds to the topological edge modes that emerge by the nonlinearity–induced topological phase transition at $w = (d - a - \alpha)/b$. In this case, the eigenvalues of the matrix in Supplementary Eq. (S13) become

$$\lambda_\pm = \frac{-d \pm \sqrt{d^2 + 4\alpha(2a - 3d + 3\alpha)}}{2\alpha}. \tag{S18}$$

We can show that these eigenvalues are real. Since the nonlinear winding number becomes $\nu_{\mathrm{NL}} = 1$ at $w > (d - a - \alpha)/b$, we expect that the dimension of the stable manifold of the fixed point at $|\psi_{\mathrm{fix}}|^2 = (d - a - \alpha)/b$ is one. However, we can confirm the breakdown of such bulk–edge correspondence by the period–doubling bifurcation as in the original nonlinear SSH model in Eqs. (4) and (5) in the main text.

We first check $|\lambda_-| > 1$ from

$$(2\alpha - d)^2 - [d^2 + 4\alpha(2a - 3d + 3\alpha)] = 8d\alpha - 8a\alpha - 8\alpha^2 < 0. \tag{S19}$$

Thus, the dimension of the stable manifold is one if $|\lambda_+| < 1$ and otherwise zero. We first show $\lambda_+ > -1$, which is equivalent to

$$d - 2\alpha < \sqrt{d^2 + 4\alpha(2a - 3d + 3\alpha)}. \tag{S20}$$

We can show this inequality from

$$(d - 2\alpha)^2 - [d^2 + 4\alpha(2a - 3d + 3\alpha)] = 8d\alpha - 8a\alpha - 8\alpha^2 < 0. \tag{S21}$$

Therefore, if and only if λ_+ is smaller than one, the absolute value of λ_+ is also smaller than one, i.e., $|\lambda_+| < 1$. We note that $\lambda_+ < 1$ is equivalent to

$$2\alpha + d > \sqrt{d^2 + 4\alpha(2a - 3d + 3\alpha)}. \tag{S22}$$

By comparing the square of the left- and right-hand sides, we obtain

$$(2\alpha + d)^2 - [d^2 + 4\alpha(2a - 3d + 3\alpha)] = 16d\alpha - 8a\alpha - 8\alpha^2 > 0. \tag{S23}$$

Therefore, $|\lambda_+|$ becomes smaller than one and thus the nonlinear winding number corresponds to the dimension of the stable manifold only in the case of $(a + \alpha)/d > 2$. At the critical parameter $(a + \alpha)/d = 2$, the dynamical system exhibits a period–doubling bifurcation. Such bifurcation induces the breakdown of the bulk–edge correspondence as in the original nonlinear SSH model in Eqs. (4) and (5) in the main text.

Stability analysis of the fixed point at $|\psi_{\mathrm{fix}}|^2 = (\alpha - a)/b$. If we consider the case of $2\alpha > d$ and $\alpha < a$, we obtain a limit cycle described as $\psi(x) = \sqrt{(\alpha - a)/b}\exp(i(\Omega x + \theta))$ whose frequency Ω satisfies $\Omega = -2\alpha\cos\theta$. This limit cycle corresponds to the topological edge mode that emerges by the nonlinearity–induced topological phase transition at $w = (\alpha - a)/b$,

and the nonlinear winding number is changed into $\nu_{\mathrm{NL}} = 2$ at the transition point. The eigenvalues of the linearized matrix in Supplementary Eq. (S13) become

$$\lambda_{\pm} = \frac{-d \pm \sqrt{d^2 + 4\alpha(2a - 3\alpha)}}{2\alpha}. \tag{S24}$$

If we consider the case that $\lambda_{\pm}$ is complex, i.e., $d^2 + 4\alpha(2a - 3\alpha) < 0$, the absolute values of the eigenvalues satisfy

$$|\lambda_{\pm}^2| = \frac{d^2 + [4\alpha(3\alpha - 2a) - d^2]}{(2\alpha)^2} = \frac{3\alpha - 2a}{\alpha} < 1. \tag{S25}$$

Therefore, the dimension of the stable manifold is two and corresponds to the nonlinear winding number. In contrast, if the eigenvalues are real, $|\lambda_{-}| < 1$ is satisfied only at $a + d/2 < 2\alpha$, which we derive from the condition that

$$(2\alpha - d)^2 - [d^2 + 4\alpha(2a - 3\alpha)] = 16\alpha^2 - 4d\alpha - 8a\alpha \tag{S26}$$

must be positive. We also confirm that $|\lambda_{+}| < 1$ is equivalent to

$$2a - 3\alpha \leq 0, \tag{S27}$$

$$\sqrt{d^2 + 4\alpha(2a - 3\alpha)} < 2\alpha + d. \tag{S28}$$

Thus, we obtain $|\lambda_{+}| < 1$ only at $a - d/2 < 2\alpha$. If we consider the case of $a + d/2 > 2\alpha$, the dimension of the stable manifold is less than two, which indicates the breakdown of the bulk–edge correspondence. This breakdown is also induced by the bifurcation of the fixed point. Therefore, the breakdown of the bulk–edge correspondence by the chaos transition is independent of the hopping range in nonlinear models.

Supplementary Note 8 - Increased number of anti–localized modes in the long–range nonlinear SSH model.

We here show that long–range hopping can also increase the number of anti–localized zero modes as well as topological localized modes. As discussed in the main text, we can regard the dimension of the stable manifold as the effective number of nonlinear zero eigenvectors. In the long–range nonlinear SSH model (Eq. (9) in the main text), nonzero fixed points can have a two–dimensional stable manifold, which implies the existence of two independent anti–localized zero modes (this also indicates the existence of two localized zero modes).

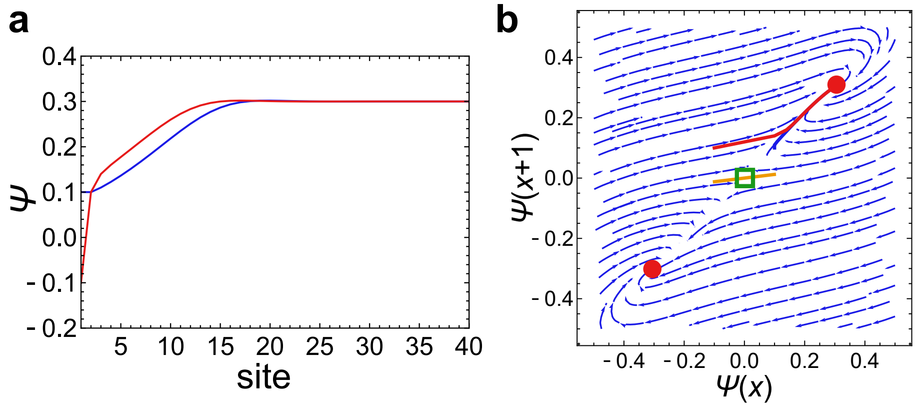

Supplementary Figure 7. **Two anti–localized zero modes in the long–range nonlinear SSH model. a** Spatial distribution of two anti–localized zero modes are presented by the red and blue polylines. By comparing the absolute value of the zero modes at the edge $x = 1$ with that in the bulk $x \to \infty$, we judge their anti–localization. These anti–localized zero modes have different values at the edge, and thus we distinguish them. We use the parameters $a = 0.11$, $b = c = 1$, $d = -1$, and $\alpha = 0.8$. **b** The flow of the nonlinear dynamical system of the long–range nonlinear SSH model (Eq. (9) in the main text) and the orbits corresponding to anti–localized zero modes. The blue curved arrows show the flow of the nonlinear dynamical system in the spatial direction. The green square and red disks are the fixed points, where the colors represent the dimensions of their stable manifolds: one (two) dimensions for the green circle (red squares). One can estimate that the red squares have two–dimensional stable manifolds from the flow around them; starting from any neighbor point, the orbit converges to the fixed point. On the other hand, the perturbation around the green fixed point diverges except for one certain direction presented by the orange line, and thus the fixed point at origin has a one–dimensional stable manifold. The blue and red curves show the orbits corresponding to the anti–localized zero modes in panel **a**. Since neither of the curves is a part of the other, they correspond to independent localized modes and indicate that the values of anti–localized modes at the edge $\Psi(1)$ have two degrees of freedom. We use the same parameters as in panel **a**.

We numerically demonstrate the existence of two anti–localized zero modes in the long–range nonlinear SSH model. We consider the parameters $a = 0.11$, $b = c = 1$, $d = -1$, and $\alpha = 0.8$. In this case, the nonlinear winding number becomes $\nu(w) = 1$ at the amplitude $w < 0.09$, and $\nu(w) = 2$ at $w > 0.09$. We calculate the spatial distribution of zero modes by

using the nonlinear dynamical system in Eq. (9) in the main text, starting from two different initial conditions $(\Psi_A(1), \Psi_A(2)) = (0.1, \pm 0.1)$. From both of the initial conditions, $\Psi_A(x)$ converges to $\Psi_A(x) \to 0.3$, which is larger than the initial amplitude 0.1 (cf. Supplementary Fig. 7a). Therefore, we obtain two anti–localized zero modes. This increase in the number of anti–localized zero modes corresponds to the two–dimensional stable manifold of the fixed point at $(\Psi_A(x), \Psi_A(x+1)) = (0.3, 0.3)$ in Supplementary Fig. 7b (two dimensions of the stable manifold are analytically shown in Supplementary Note 7).

Supplementary Note 9 - Correspondence between the nonlinear winding number and the eigenvalues of a state-dependent transfer matrix

We have also considered more general nonlinear systems whose zero modes are described by Eq. (11) in the main text. We here show that the eigenvalues of $T(g(\boldsymbol{\psi}_1(x)))$ in Eqs. (11) and (12) in the main text are related to the nonlinear winding number, which is the origin of the bulk–edge correspondence. Specifically, if we consider a fixed value of $g(\boldsymbol{\psi}(k)) = w$ independently of the wavenumber k, the nonlinear winding number

$$\nu(w) = \frac{1}{2\pi i} \int_0^{2\pi} \partial_k \log \det \left(A(w) + e^{ik} D(w) \right) dk. \tag{S29}$$

is equal to the number of the eigenvalues of $T(w) = D^{-1}(w)A(w)$ whose absolute values are less than one.

To prove the correspondence between the nonlinear winding number and the eigenvalues of the transfer matrix $T(w)$, we explicitly write down the eigenequation of $T(w)$:

$$\det(T(w) - \lambda I) = \det(D^{-1}(w)A(w) - \lambda I) = 0 \tag{S30}$$

with λ being the eigenvalue and I being an identity matrix. Since we assume that D is a regular matrix, Supplementary Equation (S30) is equivalent to

$$\det(A(w) - \lambda D(w)) = 0. \tag{S31}$$

Meanwhile, the nonlinear winding number in Supplementary Eq. (S29) is the winding number of $\det\left(A(w) + zD(w)\right)$, when we move z along a unit circle in a complex plane. According to the argument principle in complex analysis, such a winding number corresponds to the number of solutions of the algebraic equation $\det\left(A(w) + zD(w)\right) = 0$ that satisfies $|z| < 1$. By relating λ in Supplementary Eq. (S31) and z, these arguments indicate that the nonlinear

winding number is equal to the number of eigenvalues of $T(w)$ whose absolute values are less than one.

Supplementary Note 10 - Possible $\mathbb{Z}\times\mathbb{Z}$ classification in the nonlinear SSH model

If we consider the case of $b \neq c$ in the nonlinear SSH model (Eqs. (4) and (5) in the main text), edge modes localized at the left and right boundaries can appear at different critical amplitudes. To show that, we consider both left and right semi–infinite systems, $x \leq -1$ and $x \geq 1$, respectively. While the spatial distribution of zero modes in the right semi–infinite system is described by

$$\Psi_A(x+1) = -\frac{a + b|\Psi_A(x)|^2}{d}\Psi_A(x), \tag{S32}$$

(the same as Eq. (7) in the main text), that in the left semi–infinite system is described by

$$\Psi_B(x-1) = -\frac{a + c|\Psi_B(x)|^2}{d}\Psi_B(x), \tag{S33}$$

and $\Psi_A(x) = 0$. The difference between b and c leads to the different transition points in Supplementary Eqs. (S32) and (S33); the edge mode in the right semi–infinite system appears at $w = (d-a)/b$, while that in the left semi–infinite system appears at $w = (d-a)/c$. Therefore, the left edge mode and right edge mode have different critical amplitudes.

The difference in the critical amplitudes indicates the possible $\mathbb{Z}\times\mathbb{Z}$ classification in the nonlinear Su-Schrieffer-Heeger (SSH) model because the numbers of left and right edge modes can correspond to different topological invariants. In fact, one can define two nonlinear winding numbers in the nonlinear SSH model,

$$\nu_l = \frac{1}{2\pi i}\int_0^{2\pi} dk\partial_k \log(a + bw + de^{ik}), \tag{S34}$$

$$\nu_r = \frac{1}{2\pi i}\int_0^{2\pi} dk\partial_k \log(a + cw + de^{ik}), \tag{S35}$$

whose transition points are consistent with the amplitude where the left and right edge modes appear. Such a $\mathbb{Z}\times\mathbb{Z}$ classification may be analogous to that in non-Hermitian systems, where the point-gap topology of one–dimensional systems is also classified by $\mathbb{Z}\times\mathbb{Z}$ topological invariants under the existence of the sublattice symmetry [6]. However, the nonlinear SSH model considered here is a conservative system and preserves the energy in its time evolution. Therefore, nonlinearity in itself can induce non-Hermitian-like effects in the classification of topology.

We can also consider the change of the definition of w to elucidate the difference of the parameter w where the left and right edge modes appear. We discuss the possibility of different definitions of w and the extension of the bulk–edge correspondence for such w in the following section.

Supplementary Note 11 - Change of definition of the parameter w according to the nonlinearity in more general systems

In the main text, we have considered the nonlinear SSH model whose nonlinear term is proportional to $|\Psi_A(x)|^2 + |\Psi_B(x)|^2$. In general, if the nonlinear dynamics is described as

$$i\partial_t \Psi_j(x) = \sum_{l,x'} H_{jl}\left(x, x'; \sum_m |\Psi_m(x)|^2\right) \Psi_l(x'), \tag{S36}$$

where H is a matrix parametrized by $\sum_m |\Psi_m(x)|^2$, the nonlinear eigenvalue problem is equivalent to a linear eigenvalue problem under the fixed $\sum_m |\Psi_m(x)|^2 = w$. Then, one can exactly calculate the nonlinear winding number from the corresponding linear winding number.

Since the amplitude $\sum_m |\Psi_m(x)|^2 = w$ is unchanged under the time evolution in a conserved system, it is quite natural to focus on the special solutions with the fixed amplitude. However, if the strengths of nonlinear terms are not determined by the amplitude, there can be other choices of w to define the nonlinear winding number. Specifically, if the nonlinear dynamics is described as

$$i\partial_t \Psi_j(x) = \sum_{l,x'} H_{jl}\left(x, x'; g(\Psi(x))\right) \Psi_l(x'), \tag{S37}$$

where $g(\Psi(x))$ is a nonlinear function of $\Psi_j(x)$ ($j = 1, \cdots, M$ with M being the internal degree of freedom), and H is a matrix parametrized by $g(\Psi(x))$, it may be better to fix $g(\Psi(x))$ as $g(\Psi(x)) = w$ instead of the amplitude.

We first show that fixing $g(\Psi(x)) = w$ enables us to calculate the nonlinear eigenequation (Supplementary Eq. (S37)). The wavenumber–space description of the nonlinear eigenequation corresponding to Supplementary Eq. (S37) becomes

$$E(k)\psi_j(k) = \sum_{l} H_{jl}\left(k; g(\psi(k))\right) \psi_l(k). \tag{S38}$$

If we focus on special solutions with $g(\psi(k)) = w$ being fixed independently of k, the right-hand side reads $\sum_l H_{jl}\left(k; w\right) \psi_l(k)$. Therefore, the nonlinear eigenvalue problem becomes

equivalent to the linear eigenvalue problem parametrized by w and k, and thus one can calculate the eigenvalues and eigenvectors by solving linear equations. We note that the multiple of the obtained eigenvector $\psi(k)$ is also an eigenvector because of the linearity. Thus, the final step of the calculation of the nonlinear eigenvector is to find the constant $c(k)$ that satisfies

$$g(c(k)\psi(k)) = w. \tag{S39}$$

Such $c(k)$ can be absent for certain $\psi(k)$ and w, while one can guarantee the existence of $c(k)$ satisfying Supplementary Eq. (S39) if the nonlinear function has desirable properties. Specifically, if g is a continuous function, one can always find $c(k)$ for w in the range of $\max_c \min_\psi g(c\psi) \leq w \leq \min_c \max_\psi g(c\psi)$.

We can also discuss the bulk–edge correspondence of the winding number under the fixed $f(\Psi(x)) = w$. In particular, if we focus on the generalized nonlinear SSH model,

$$i\partial_t \Psi_A(x) = (a + g(\Psi_A(x), \Psi_B(x)))\Psi_B(x) + d\Psi_B(x-1), \tag{S40}$$

$$i\partial_t \Psi_B(x) = (a + g(\Psi_A(x), \Psi_B(x)))\Psi_A(x) + d\Psi_A(x+1), \tag{S41}$$

where $g(\Psi_A(x), \Psi_B(x))$ is a real nonlinear function of $\Psi_A(x)$ and $\Psi_B(x)$, we can confirm the bulk–edge correspondence in a similar sense to that in the original nonlinear SSH model (Eqs. (4) and (5) in the main text). First, we can calculate the nonlinear winding number as $\nu(w) = \int_0^{2\pi} dk \partial_k \log(a + w + e^{ik})/(2\pi i)$, which is $\nu = 1$ (resp. $\nu = 0$) in the case of $a + w < d$ (resp. $a + w > d$). Then, we derive the nonlinear dynamical system describing the spatial distribution of zero modes in the semi–infinite system,

$$\Psi_A(x+1) = -\frac{a + g(\Psi_A(x), 0)}{d}\Psi_A(x). \tag{S42}$$

When $a + g(\Psi_A(x), 0)$ is smaller (larger) than d, $|\Psi_A(x+1)|$ becomes smaller (larger) than $|\Psi_A(x)|$. This implies that there is a fixed point at $\Psi'_A(x)$ satisfying $a + g(\Psi'_A(x), 0) = d$, and it can be stable if $\nu(w)$ is one for at $w = g(\Psi'_A(x) + \delta, 0)$ and zero at $w = g(\Psi'_A(x) - \delta, 0)$ with δ being a sufficiently small constant. If $\Psi'_A(x)$ is a stable fixed point, there are nonlinear edge modes with the initial amplitude $\Psi_A(1)$ satisfying $\nu(g(\Psi_A(1), 0)) = 1$, which indicates the bulk–edge correspondence of the nonlinear winding number. However, Supplementary Equation (S42) can exhibit bifurcations, and $\Psi'_A(x)$ can be an unstable fixed point after the bifurcations. Therefore, the bulk–edge correspondence is only valid for sufficiently weak nonlinearity as in the original nonlinear SSH model.

Supplementary References

[1] Bisianov, A., Wimmer, M., Peschel, U., & Egorov, O. A. Stability of topologically protected edge states in nonlinear fiber loops. *Phys. Rev. A* **100,** 063830 (2019).

[2] Sone, K., Ezawa, M., Ashida, Y., Yoshioka, N., & Sagawa, Nonlinearity-induced topological phase transition characterized by the nonlinear chern number. *Nat. Phys.* **20,** 1164–1170 (2024).

[3] Mayer-Kress, G. & Haken, H. The influence of noise on the logistic model. *J. Stat. Phys.* **26,** 149–171 (1981).

[4] Tuloup, T., Bomantara, R. W., Lee, C. H., & Gong, J. Nonlinearity induced topological physics in momentum space and real space. *Phys. Rev. B* **102,** 115411 (2020).

[5] Ma, F. et al., Nonlinear Topological Mechanics in Elliptically Geared Isostatic Metamaterials. *Phys. Rev. Lett.* **131,** 046101 (2023).

[6] Kawabata K., Shiozaki K., Ueda M., & Sato M. Symmetry and Topology in Non-Hermitian Physics. *Phys. Rev. X* **9,** 041015 (2019).
